# Forest terrains influence walking kinematics among indigenous Tsimane of the Bolivian Amazon

**DOI:** 10.1017/ehs.2022.13

**Published:** 2022-04-22

**Authors:** Nicholas B. Holowka, Thomas S. Kraft, Ian J. Wallace, Michael Gurven, Vivek V. Venkataraman

**Affiliations:** 1 Department of Anthropology, University at Buffalo, Buffalo, NY, USA; 2 Department of Anthropology, University of Utah, Salt Lake City, UT, USA; 3 Department of Anthropology, University of California-Santa Barbara, Santa Barbara, CA, USA; 4 Department of Anthropology, University of New Mexico, Albuquerque, NM, USA; 5 Department of Anthropology and Archaeology, University of Calgary, Calgary, Canada

**Keywords:** Locomotor ecology, bipedalism, barefoot walking, forager–horticulturalist

## Abstract

Laboratory-based studies indicate that a major evolutionary advantage of bipedalism is enabling humans to walk with relatively low energy expenditure. However, such studies typically record subjects walking on even surfaces or treadmills that do not represent the irregular terrains our species encounters in natural environments. To date, few studies have quantified walking kinematics on natural terrains. Here we used high-speed video to record marker-based kinematics of 21 individuals from a Tsimane forager–horticulturalist community in the Bolivian Amazon walking on three different terrains: a dirt field, a forest trail and an unbroken forest transect. Compared with the field, in the unbroken forest participants contacted the ground with more protracted legs and flatter foot postures, had more inclined trunks, more flexed hips and knees, and raised their feet higher during leg swing. In contrast, kinematics were generally similar between trail and field walking. These results provide preliminary support for the idea that irregular natural surfaces like those in forests cause humans to alter their walking kinematics, such that travel in these environments could be more energetically expensive than would be assumed from laboratory-based data. These findings have important implications for the evolutionary energetics of human foraging in environments with challenging terrains.

## Graphical abstract

**Figure:**
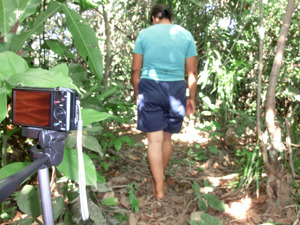

**Social media summary:** Tsimane individuals use more crouched legs and inclined trunks to walk through forests compared to fields and trails.

## Introduction

Bipedal locomotion is one of the defining characteristics of humans. Metabolic studies indicate that a key advantage of human bipedalism is greater walking economy than in other primates, including our closest living relatives, chimpanzees (Nakatsukasa et al., 2004; Sockol, Raichlen, & Pontzer, 2007). This means we use relatively less energy to move a given distance than other primates. The distinct kinematic features of human bipedal walking that contribute to this enhanced economy include the use of extended hip and knee joints, narrow step widths, and heel-first foot strike postures (O'Neill, Demes, Thompson, & Umberger, 2018; Pontzer, Raichlen, & Sockol, 2009; Thompson, O'Neill, Holowka, & Demes, 2018; Webber & Raichlen, 2016).

While these energy-saving kinematics are commonly described as typical characteristics of human walking (Simoneau, 2002), our knowledge of ‘standard’ human walking kinematics and energetics is based almost entirely on laboratory studies of individuals walking on treadmills or flat, even walkways. These surfaces differ considerably from the ‘natural’ terrains that exist outside of the laboratory, particularly those that humans would have commonly travelled on before roads and paved surfaces became common. The surfaces of many natural terrains, such as rocky ground, savannah brushland and forest floors, are characterised by uneven textures, three-dimensional impediments (e.g. rocks, roots, vegetation) and varying firmness that could cause individuals to alter their ‘standard’ kinematics to maintain balance and/or avoid stepping on hazardous objects (Matthis, Yates, & Hayhoe, 2018; Venkataraman et al., 2018). However, relatively few studies have investigated human walking on such surfaces.

Recently, some laboratory-based studies have attempted to simulate natural terrain walking by having subjects walk on artificial, ‘irregular’ surfaces consisting of regularly spaced obstacles like short blocks, compliant materials or loose rocks (reviewed in Hawkins, Clark, Balasubramanian, & Fox, 2017). These surfaces are typically designed to prevent participants from achieving consistent footholds from step to step, and/or to reduce the stability of the underlying surface. Generally, these studies have found that, when compared with walking on regular (flat, even) surfaces, participants use more variable stride characteristics like step length and width (e.g. Kent, Sommerfeld, Mukherjee, Takahashi, & Stergiou, 2019; Menant, Steele, Menz, Munro, & Lord, 2011; Voloshina, Kuo, Daley, & Ferris, 2013), use flatter foot postures at initial contact and higher foot clearance during leg swing (Gates, Wilken, Scott, Sinitski, & Dingwell, 2012; e.g. Schulz, 2011), and have greater leg muscle activation (e.g. Blair, Lake, Ding, & Sterzing, 2018; Voloshina et al., 2013) when walking on irregular surfaces. These adjustments are hypothesised to help individuals maintain balance on uneven footing and avoid tripping. One study found that, because of such factors, participants exhibited a 28% increase in rate of energy expenditure when walking on a custom-designed uneven treadmill surface compared with a standard, even treadmill surface (Voloshina et al., 2013). However, few studies have investigated walking kinematics or energetics outside of a laboratory in complex three-dimensional environments with naturally irregular surfaces. Walking outdoors on relatively simple natural surfaces (e.g. grass, gravel, woodchips) can increase walking costs by up to 27% owing to factors such as stride variability and height of swing foot clearance (Kowalsky, Rebula, Ojeda, Adamczyk, & Kuo, 2021), whereas more complex (albeit poorly defined) natural surfaces like ‘heavy brush’ and ‘swampy bog’ may increase metabolic rates by as much as 50–80% compared with flat surface walking (Soule & Goldman, 1972). Several recent studies of walking on rocky terrains found the individuals significantly reduced their step lengths to achieve more stable foot placement when compared with walking on flat, even surfaces (Gast, Kram, & Riemer, 2019; Matthis et al., 2018), which more than doubled their walking costs (Gast et al., 2019), suggesting that naturally irregular terrains may interrupt standard gait kinematics more than those simulated in laboratories.

These laboratory- and field-based studies of irregular surface walking have major implications for the energetic demands of human foraging from an evolutionary perspective. Hunting and gathering has been the sole mode of subsistence for most of our species’ existence, and recent hunter–gatherer populations have been found to walk 6–19 km per day (Leonard & Robertson, 1997a; Wood et al., 2021), much of which is likely to be on irregular surfaces. However, the irregular surface studies described thus far have used participants from post-industrial societies who may not walk on natural terrains regularly, and therefore may not use the kinematic strategies and associated neuromuscular activation patterns necessary to navigate these terrains most efficiently. Perhaps more significantly, the individuals in these studies wore sophisticated, modern footwear such as athletic shoes and boots, which have not been available to our species for most of its existence (Tenner, 2003) and probably affect the way we walk on challenging surfaces. Therefore, to understand the energetic consequences of walking on irregular surfaces in human foraging, research with individuals living in close association with undeveloped natural environments is necessary.

Recently, we investigated how walking in forest vs. open environments affected step length and speed in two rainforest-dwelling populations (Venkataraman et al., 2018): the Batek from Peninsular Malaysia and the Tsimane of the Bolivian Amazon. We found that in both populations, participants decreased their average step length in the forest when compared with walking on even, open field surfaces, and we argued that this adjustment was probably made because step length is constrained by the need to find stable foot placement and avoid obstacles like tree roots and other vegetation. Reducing step length below that preferred on regular surfaces should increase the energy costs associated with walking on natural terrains like those in forests (Gast et al., 2019; Umberger & Martin, 2007), but as with laboratory-based studies using irregular surfaces, the participants in our study may have made other adjustments to their kinematics that could have further influenced walking energetics. For instance, ethnographers have observed that humans from populations native to dense rainforests walk with crouched postures and high stepping gaits when in the forest in order to avoid both head-level (e.g. branches) and foot-level (e.g. roots) vegetation that could cause harm (Evans, 1937; Garvan, 1964; Schebesta, 1928; Turnbull, 1986). Such kinematic adjustments would almost certainly increase walking costs, but no study has actually quantified lower limb or joint kinematics during walking on irregular surfaces in challenging natural environments like forests.

While walking through unbroken forest is certainly necessary during foraging among forest-dwelling hunter–gatherers, people everywhere make and utilise trails, which could offer several advantages during walking. First, well-maintained human-made trails could help mitigate travel costs by allowing individuals to use more standard, energetically optimal walking kinematics. Second, trails in natural environments can protect against the potentially severe costs of getting lost during travel, thereby allowing for safer movement across greater ranges. Third, trails allow for communication via markings along standardised routes. While there are few studies on human trail use, research on other animals, typically conducted using camera traps, has indicated substantial energetic benefits of trail use. Carnivores and large-bodied predators use trails more than other animals, including their prey (e.g. small ungulates; Cusack et al., 2015; Kays et al., 2011), and use large day ranges to hunt (Carbone, Cowlishaw, Isaac, & Rowcliffe, 2005). In this context, using trails probably decreases the costs of locomotion and facilitates higher travel speeds. Trails may also aid patrolling behaviour in territorial species (Cusack et al., 2015), although a study of Tai chimpanzees found that they rarely use paths, including while on patrols (Jang, Boesch, Mundry, Ban, & Janmaat, 2019). Given that humans have longer day ranges than other primates (Pontzer & Kamilar, 2009), the use of trails in dense environments (such as forests) is expected to save energy and decrease travel time.

To begin to explore how natural terrains and trails affect walking in humans that engage in foraging, we measured video-based lower limb kinematics in Tsimane individuals walking in unbroken forest, in an open field and on a forest trail. The Tsimane are a forager–horticulturalist society from the lowland Amazon of Bolivia (Gurven et al., 2017), and make an ideal study population because they walk through dense forest regularly for common activities such as food gathering, hunting and travelling to horticultural fields or to visit relatives in other villages. Additionally, they often do so barefoot or in simple, minimal footwear. Video-based kinematics impose certain limitations that we will discuss later, and our sample size was limited by access to willing participants in a remote setting, so we consider this investigation of forest walking to be preliminary. Nevertheless, we believe these data are vital to establishing and refining hypotheses for future research.

We predicted that participants in our study would make similar kinematic adjustments when walking in the forest to those observed during irregular surface walking in laboratory-based studies. Specifically, we predicted that, relative to walking in open fields, during unbroken forest walking participants would contact the ground with their feet less angled and therefore ‘flatter’ relative to the ground surface, and that they would lift their feet higher during leg swing. Based on previous ethnographic observations, we also predicted that participants would walk with more flexed lower limb joints and more inclined (forward-leaning) trunks in the forest. Additionally, following our previous findings (Venkataraman et al., 2018), we predicted that participants would use less protracted lower limbs at foot contact during forest walking in order to shorten step length. Lastly, we predicted that, when walking through the forest on human-made trails, these individuals would employ lower limb kinematics similar to those used when walking in an open field.

## Methods

### Study sample

We collected data from 21 Tsimane individuals (males, *N* = 10; females, *N* = 11) from the Jämsi Bayedye village located in the Beni Department of Bolivia (Table 1). Jämsi Bayedye is a semi-market integrated community along the Maniqui River consisting of a mix of traditional and concrete houses surrounded by horticultural fields and both primary and secondary rainforest. Participants ranged in age from 15 to 70 years old. None reported any injury that would affect walking, and none exhibited any visible problems with their gait. All participants in the study provided informed consent and were compensated for their participation. All study procedures were approved by the institutional review board of the University of California, Santa Barbara.

**Table 1.** Participant sample size and anthropometrics. Anthropometrics reported as mean (standard deviation).

Sex	*N*	Value	Age (years)	Weight (kg)	Height (cm)	Leg length (cm)
Female	11	Mean (SD)	26.5 (12.9)	60.2 (11.7)	152.7 (4.9)	77.9 (3.3)
		Range	15–45	46.8–87.2	142–156.8	73–84
Male	10	Mean (SD)	38.4 (19.5)	64.5 (7.1)	162.5 (4.4)	82.4
		Range	17–70	53.1–74.2	156.2–168.6	77–88

### Walking conditions

**Figure 1.**
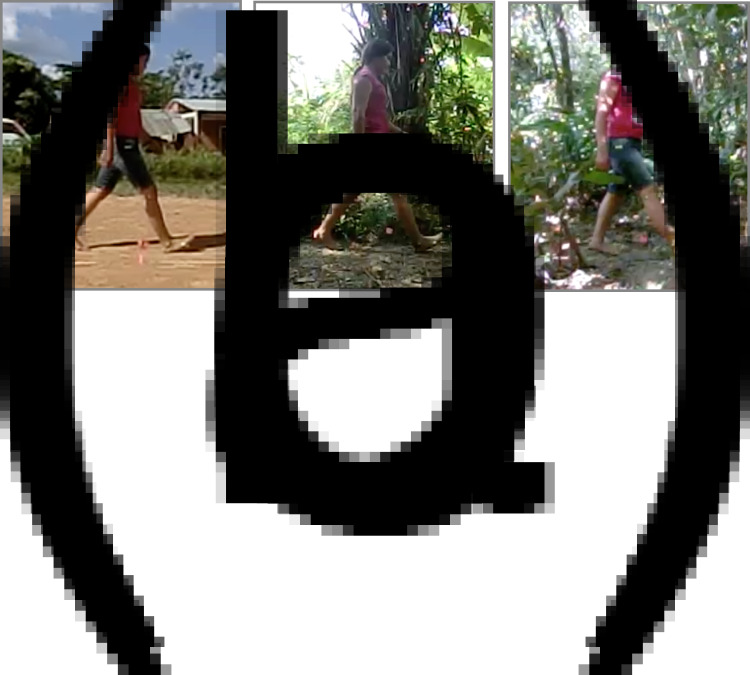
Walking conditions investigated in this study: (a) open field; (b) forest trail; and (c) unbroken forest.

We recorded participants walking in three conditions: through an open field, on a trail through the forest and along a transect through the forest understory. For the open field trials, participants walked on a flat, even surface consisting of dry, hard-packed dirt that is used as a soccer field in the village. In terms of stiffness and evenness, this surface was assumed to be similar (but not identical) to the surfaces commonly used in laboratory-based walking studies. For the forest trail trials, they walked on a linear segment of a relatively flat human-made trail through the forest. Like many trails utilised by the Tsimane, this trail had been created by cutting back vegetation from a pre-existing animal trail, had been expanded over many years of repeated walking and was regularly maintained by clearing vegetation with machetes. Lastly, for the unbroken forest understory trials, participants walked through a linear 14 m transect of unbroken secondary forest. For all conditions, evenly spaced flags were used to mark the walking path. We instructed participants to walk at a speed that they would consider to be a comfortable pace during foraging. Participants were recorded using a Casio EX-ZR100 camera (Casio Computer Co. Ltd.) mounted on a tripod that was set up on an even, level surface, set at a capture rate of 240 frames per second. The camera was positioned perpendicular to the intended direction of travel to capture sagittal plane motion (Figure 1), and was set such that the horizontal plane of the camera view was parallel to the walking route marked by the flags, which was used to define the *x*-axis of the real-world coordinate system (with the *y*-axis being perpendicular to this). For all open trials, the camera was positioned 5 metres from the walking route. For all forest and trail trials, the camera was positioned 3 metres from the walking route such that one or two full strides could be captured within the camera's viewing window. The segment of the forest transect captured in this window was selected because it did not include any particularly large obstacles that would cause major alterations in gait kinematics, but still included smaller impediments like vegetation and roots that we deemed to be representative of the overall footing across the walking transect. In all conditions, participants walked the same routes by following the flags, and therefore participants’ bodies were roughly the same distance from the camera in all recordings. During all walking trials participants walked barefoot at preferred speeds. Tsimane individuals frequently walk barefoot in their settlements and prefer to walk barefoot when in the forest. We recorded three trials for each participant in each condition.

### Kinematics

**Figure 2.**
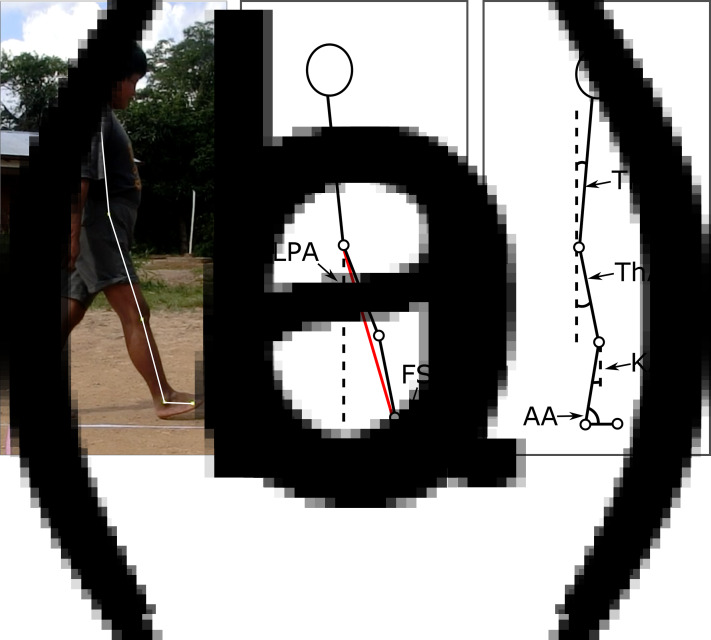
Marker positions and body segments analysed (a), and angles measured at foot strike (b) and midstance (c). Dashed lines represent the vertical plane of the camera and solid lines indicate trunk and lower limb segments. LPA, Leg protraction angle; FSA, foot strike angle; TrA, trunk inclination angle; ThA, thigh angle; KA, knee angle; and AA, ankle angle. The FSA value used in analysis was calculated by deducting the FSA measured when the foot was flat on the ground from the FSA measured at foot strike. The AA values used in analysis were calculated by deducting the AA measured at foot strike and midstance from the AA measured when the foot was flat on the ground and the shank was perpendicular to the ground.

Prior to recording walking trials, we affixed tape markers to the legs and feet of participants at the following anatomical landmarks to indicate lower limb joint positions: the greater trochanter (‘hip marker’), the lateral epicondyle of the knee (‘knee marker’) and the lateral malleolus of the ankle (‘ankle marker’). We also placed a marker on the fifth metatarsal head to indicate the distal end of the foot (‘forefoot marker’) (Figure 2). All anatomical landmarks were determined by manual palpation. We had to affix the hip marker to the participant's clothes overlying the greater trochanter, but the other markers were affixed directly on the participant's skin.

After data collection, we inspected all trial videos to determine the suitability for analysis. Trials were kept for analysis based on two criteria. First, the participant appeared to maintain constant forward velocity without any abrupt changes in lower limb trajectories owing to, for instance, slips or obstacle avoidance. We included this criterion because the goal of this study was to understand the general changes in gait kinematics, not the acute effects of obstacles. The second criterion was that all markers were visible at both foot strikes and midstance of the selected stride. A small number of forest trials had to be removed because they failed the first criterion, but many had to be removed owing to the second, specifically because foliage obstructed views of markers. Ultimately, one to three trials were kept per walking condition per participant, except for three participants for whom there were no analysable forest trials. Trials that were kept for analysis were digitised in MATLAB (The Mathworks, Natick, MA, USA) using DLTdV software (Hedrick, 2008). We digitised one stride per walking trial. To test our predictions, we digitised marker positions at first foot strike (beginning of the stride), and midstance, which we defined as the video frame in which the swing leg foot first passed behind the stance leg. To calculate trunk inclination angle (see below), we also digitised the apex of the shoulder at midstance. To calculate foot strike angle and ankle angle (see below), we digitised marker positions at the first video frame after foot strike in which the foot was flat on the ground *and* the shank was perpendicular to the ground. Additionally, we digitised the ankle position at the video frame where the ankle marker reached its maximum height during swing phase and the hip marker position at the second foot strike. Digitised coordinate data were exported for further analysis in custom-written MATLAB scripts.

At the first foot strike we calculated leg protraction as the angle of the line connecting the hip and ankle markers relative to the vertical plane of the camera view (Figure 2b). We calculated foot strike angle as the angle of the foot (the line connecting the ankle and forefoot markers) relative to the horizontal plane of the camera view at foot strike. To standardise foot strike angle across participants and conditions for analysis, we calculated foot angle relative to the horizontal when the foot was flat on the ground and deducted this angle from the foot strike angle measurement described above. At midstance, we calculated trunk inclination angle as the angle of the line connecting the hip marker and the shoulder relative to the vertical plane (Figure 2c). We also calculated midstance thigh angle as the angle of the line connecting the hip and knee markers relative to the vertical plane. We calculated midstance knee angle as 180° minus the angle between the thigh and shank (knee marker to ankle marker). Finally, we calculated ankle angle as the angle between the shank and foot segments at both foot strike and midstance. For these calculations, we first measured the ‘neutral position’ ankle angle at a frame in the stance phase where the foot was flat on the ground and the shank was perpendicular to the ground. To set this position as 0°, we deducted ankle angle values measured at foot strike and midstance from the neutral position value, such that positive ankle angle values corresponded to dorsiflexion and negative values corresponded to plantarflexion relative to the ‘neutral position’.

For all distance calculations, we calibrated our measurements based on the known distance between flags in the camera view (2 metres for open trials, 1 metre for forest and trail trials). We calculated stride length as the horizontal distance travelled by the hip marker from first foot strike to second foot strike, stride duration as the time between foot strikes and walking velocity as stride length divided by stride duration. Stride frequency was calculated as 1/stride duration. We calculated maximum foot height as the vertical position of the ankle marker at its maximum height during swing phase, minus the vertical position of the forefoot marker when the foot was flat on the ground. We used the ankle marker for the maximum foot height calculation because, unlike the forefoot marker, its vertical position would not be affected by ankle angle during swing.

### Statistics

All statistical tests were carried out using R version 3.6.1 (R Core Team, 2019). First, we performed Shapiro–Wilk tests on each variable to determine if the variables were normally distributed. Only stride frequency required natural log transformation for normality. We used the ‘lme4’ package (Bates, Mächler, Bolker, & Walker, 2015) to estimate linear mixed effects models for each variable, with walking condition (‘open’, ‘trail’, ‘forest’) and walking velocity included as fixed effects and participant identity as a random effect. We also estimated two additional linear mixed effects models to assess the possible determinants of foot strike angle. In these models, foot strike angle was the response variable, either leg protraction angle or ankle angle was the independent variable and participant identity was a random effect. Finally, to assess the possible effects of participant height on trunk lean and joint flexion during forest transect walking, we estimated linear mixed effects models from just the forest transect steps. In these models, midstance trunk inclination, hip flexion and knee flexion were the response variables, participant height and velocity were independent variables, and participant identity was a random effect.

For all models, we inspected residual plots and q–q plots to assess model residual homoscedasticity and normality, respectively. For all variables these criteria were satisfied, and therefore we performed likelihood ratio tests to test for significant model effects. For models where significant differences in walking conditions were detected, we used the ‘lsmeans’ package (Lenth, 2016) to conduct post-hoc pairwise contrasts between walking conditions, with a Holm–Bonferroni *p*-value correction. For all tests, we used an alpha value of 0.05 to assess statistical significance.

## Results

We analysed a total of 127 trials, including 49 open trials, 47 trail trials and 31 forest trials. Average values for all kinematic variables are presented in Table 2 and the results of statistical tests are presented in Table 3. Participants walked 13% faster on average on the trail than in the open (*p =* 0.0006), and 16% faster on average on the trail than in the forest (*p =* 0.0006), but there was no significant difference between open or forest walking velocity. Controlling for velocity, participants had significantly different stride lengths and frequencies on all surfaces (*p* < 0.0001). Participants used the highest stride frequencies and shortest strides in the open, and the lowest stride frequencies and longest strides in the forest.

**Table 2.** Gait variables measured in this study. Results reported as mean (standard deviation).

		Condition		
Gait Event	Variable	Open	Trail	Forest
General	Velocity (m/s)	1.23 (0.23)	1.39 (0.21)	1.2 (0.21)
	Stride frequency (Hz)	0.98 (0.09)	0.94 (0.11)	0.79 (0.1)
	Stride length (l)^a^	1.56 (0.19)	1.85 (0.18)	1.89 (0.25)
Foot strike	Leg protraction (deg)	23.4 (3.5)	25.5 (3.5)	26.6 (2.7)
	Foot strike angle (deg)	19.7 (8.9)	18.1 (7.9)	12.2 (8.4)
Midstance	Trunk angle (deg)	−2.01 (4.3)	−2.27 (3.9)	3.48 (7.8)
	Thigh angle (deg)	3.52 (5.7)	7.75 (6.5)	12.5 (5.6)
	Knee Angle (deg)	12.9 (6.6)	15.8 (7.5)	20.7 (11.1)
	Ankle angle (deg)	7.21 (8.3)	7.42 (6)	8.73 (8)
Leg swing	Max ankle height (l)^a^	0.23 (0.02)	0.29 (0.03)	0.34 (0.06)

a Unit is in leg lengths (l), measured as the height of the greater trochanter during standing.

**Table 3.** Results of statistical tests for effects of walking condition on kinematic variables.

		Likelihood ratio test^a^		Post-hoc paired tests^b^		
Variable		Walking condition	Velocity	Open–trail	Open–forest	Trail–forest
Velocity	p	<0.0001	—	0.0006	0.6	0.0006
	stat	18.8	—	−3.8	0.5	3.8
Stride frequency	p	<0.0001	<0.0001	<0.0001	<0.0001	<0.0001
	stat	111.4	104.1	6.9	13.2	6.3
Stride length	p	<0.0001	<0.0001	<0.0001	<0.0001	<0.0001
	stat	99.5	108.0	−6.3	−12.4	−6.1
Leg protraction	p	<0.0001	<0.0001	0.043	<0.0001	0.002
	stat	28.0	22.6	−2.1	−5.5	−3.4
Foot strike angle	p	0.0003	0.002	0.084	0.0002	0.084
	stat	16.5	9.7	2.1	4.1	2.1
Trunk angle	p	<0.0001	0.055	0.25	<0.0001	<0.0001
	stat	30.2	3.7	1.2	−4.8	−5.4
Thigh angle	p	<0.0001	0.52	0.0001	<0.0001	0.001
	stat	48.5	0.41	−4.2	−7.5	−3.3
Knee angle	p	<0.0001	0.052	0.16	<0.0001	0.004
	stat	21.5	3.8	−1.4	−4.8	−3.2
Ankle angle	p	0.36	0.072	—	—	—
	stat	2.0	3.2	—	—	—
Max ankle height	p	<0.0001	0.055	<0.0001	<0.0001	<0.0001
	stat	107.4	3.7	−6.2	−12.7	−6.5

a Tests conducted on model variance from linear mixed effects models, which included velocity as a covariate. Statistical results for fixed effect (walking condition) and covariate (velocity) reported. Test-statistic for these tests is chi-squared.

b Post-hoc paired comparison tests for differences in walking conditions. A Holm–Bonferroni correction was used to correct *p*-values.

**Figure 3.**
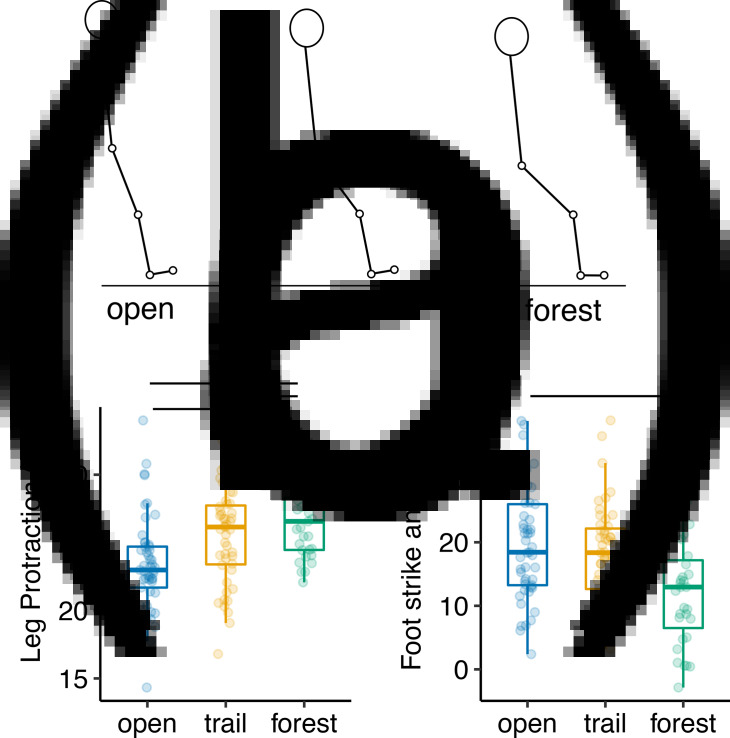
Results for kinematic variables at foot strike during walking in the open field, on the forest trail and through unbroken forest. (a) Representative segment angles at foot strike in all three conditions based on marker positions (see Figure 2). Approximate segment angles depicted here are slightly exaggerated to demonstrate differences between conditions. (b, c) Leg protraction angle and foot strike angle, respectively. Points represent individual steps, boxes represent interquartile ranges, middle bars represent median values and whiskers extend to the data point ± 1.5× the interquartile range. Bars over boxes indicate significant (*p* < 0.05) differences between conditions.

**Figure 4.**
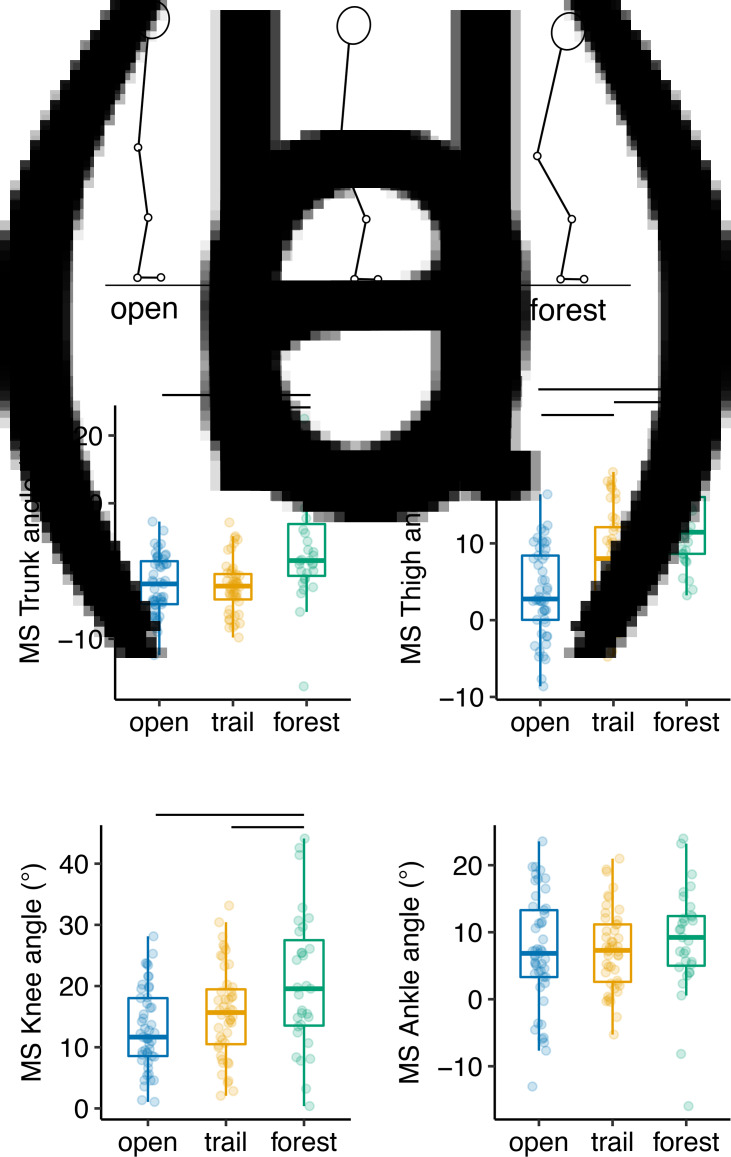
Results for kinematic variables at midstance (MS) during walking in the open field, on the forest trail and through unbroken forest. (a) Representative segment angles at foot strike in all three conditions based on marker positions (see Figure 2). Approximate segment angles depicted here are slightly exaggerated to demonstrate differences between conditions. (b) Trunk inclination angle; (c) thigh angle; (d) knee angle; and (e) ankle angle. Points represent individual steps, boxes represent interquartile ranges, middle bars represent median values and whiskers extend to data point ± 1.5× the interquartile range. Bars over boxes indicate significant (*p* < 0.05) differences between conditions.

When walking in the unbroken forest, participants contacted the ground with 14% more protracted legs (*p* < 0.0001) and 38% lower foot strike angles (*p =* 0.0002) on average than when walking in the open field (Figure 3). This means that, in the forest, participants landed with their legs further out in front of their bodies, and with their feet in ‘flatter’ postures, in contrast to the more heel-first foot strike postures used in the open. Participants also had 4% more protracted legs at foot strike on average in the forest than they did on the trail (*p =* 0.002). Participants had more inclined trunks (*p* < 0.0001), flexed knees (*p* < 0.005) and higher thigh angles (*p* < 0.002) at midstance during forest walking compared with the other walking conditions (Figure 4). The latter result indicates the use of more flexed hip joints at midstance in the forest. Additionally, among forest walking steps, each of these variables was significantly, positively associated with participant height, meaning that taller participants tended to flex their hip (*p =* 0.002) and knee joints (*p =* 0.01) more and incline their trunks further forward (*p =* 0.013) when walking in the forest. However, participants used similar midstance ankle angles in all walking conditions (*p =* 0.36). Finally, participants had the highest maximum ankle heights when walking in the forest (*p* < 0.0001), meaning that they lifted their feet 17–48% higher off the ground on average during swing phase in the forest than in the other conditions.

Overall, participants used similar lower limb kinematics when walking on the trail and walking in the open field. However, participants contacted the ground with 9% more protracted legs on average on the trail (*p =* 0.043) (Figure 3), and also had higher midstance thigh angles (*p =* 0.0001) and thus more flexed hips on the trail than in the open (Figure 4). Additionally, participants had 26% higher maximum foot heights on average during swing phase on the trail than they did when walking in the open (*p* < 0.0001).

**Figure 5.**
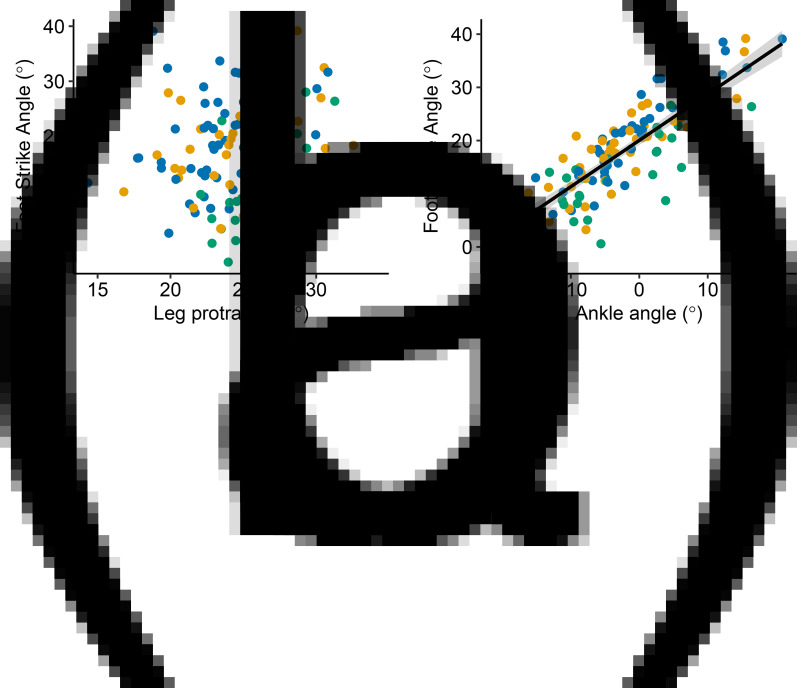
Relationships between foot strike angle and leg protraction angle (a) and ankle angle (b). Points represent individual steps during walking in the open field (blue), on the forest trail (yellow) and through unbroken forest (green). The solid line in (b) is the ordinary least squares regression line that describes the relationship between ankle angle and foot strike angle, and the shaded region represents the 95% confidence interval for this regression model.

Across conditions, we found that ankle angle had a significant effect on foot strike angle (*p* < 0.0001), but leg protraction angle did not (*p* = 0.22) (Figure 5).

## Discussion

With this study we present a preliminary investigation of how humans adjust their kinematics when walking on irregular surfaces in natural environments. We measured walking kinematics on three terrain types representing a spectrum of surface irregularity (open field, flat trail, forest understory) in Tsimane participants who are accustomed to walking on these surfaces barefoot as part of their daily livelihood. Our predictions, based on ethnographic observation and laboratory studies, were broadly supported: participants walked with flatter foot strikes, more inclined trunks, more flexed lower limb joints and higher maximum foot heights during leg swing when walking in unbroken forest compared with open fields. We also found mixed support for our prediction that participants would use similar lower limb kinematics when walking on the trail and when walking in the open field. Altogether, these results support the hypothesis that when walking on irregular terrains with natural impediments (such as those in the forest), humans adjust their kinematics away from those used on smooth, even surfaces that would be considered optimal for walking economy.

In the forest, participants tended to land with lower foot strike angles than when in the open or on the trail, meaning that their feet were oriented closer to parallel with ground when contact was made, resulting in a flatter foot contact pattern than the discrete heel strikes characteristic of human walking on even surfaces (O'Neill et al., 2018; Webber & Raichlen, 2016). These results were similar to those of laboratory studies where humans used flatter foot contacts while walking over loose rocks (Gates et al., 2012) and artificial uneven surfaces (Voloshina et al., 2013). The use of a flatter foot contact and absence of a clear heel strike may affect walking economy, as it may reduce the total distance travelled by the body's centre of mass during a given step (Webber & Raichlen, 2016) and/or increase the amount of mechanical work needed to accelerate the body's centre of mass between steps (Adamczyk & Kuo, 2013; Holowka & Lieberman, 2018). Across the different surfaces investigated in this study, foot strike angle was associated with ankle angle but not leg protraction angle. Thus, it appears that a flatter foot strike posture is under intentional control at the ankle, and is not just determined by more proximal positioning of the leg at foot contact. Gates et al. (2012) suggested that a flatter foot contact might be used on loose rocks or slippery surfaces to increase the foot's contact area with ground, and thus its coefficient of friction, thereby preventing slipping. The forest floor in the transect used in this study was not slippery, but it did present hazards such as tree roots that could cause tripping or foot injury. Therefore, we propose an alternative explanation for the use of flatter foot contacts in the forest: the plantar surface of the foot, even when thickly callused, maintains high tactile sensitivity that allows individuals to sense the stability and safety of the ground below their feet (Holowka et al., 2019; Wallace, Koch, Holowka, & Lieberman, 2018). Thus, by landing with a flatter foot, more of the plantar surface of the foot contacts the ground earlier in the step, providing more tactile sensation to the surface of the foot and thereby providing more sensory feedback that will allow for postural adjustments before the leg assumes full weight-bearing. This hypothesis requires further testing, but we note that study participants stated a preference for walking barefoot over using footwear in the forest, perhaps because of the additional sensory feedback afforded by the former.

Compared to walking in the open condition, participants walking in the forest had more flexed thighs and knee joints and used greater trunk inclination at midstance. There were no differences in midstance ankle joint angles between conditions, probably because limiting ankle dorsiflexion would help prevent the body's centre or mass from moving too far in front of the foot owing to trunk inclination while walking in the forest (Figure 4a). A more inclined trunk could conceivably serve two purposes in a dense forest: first, it could help individuals avoid head-level objects like branches, and second, it could bring the head closer to the ground and thereby improve an individual's ability to see the footing ahead. Matthis et al. (2018) found that, when walking on rocky terrain, people maintain their gaze on the ground in front of them roughly 95% of the time to plan foot placement two to three steps ahead of the current one. It is likely that our participants used a similar strategy to afford consistent gait dynamics in the cluttered forest understory, and inclining the trunk may have aided in this purpose, especially given that the forest floor is darkened by foliage that blocks sunlight. Regardless of the reason, trunk inclination could have a broader effect on gait biomechanics as it shifts the body's centre of mass forward slightly, potentially increasing external moments at the hip joint. The use of more flexed hip and knee postures in the forest could also increase external joint moments, thereby increasing the volume of active muscle and accordingly energy expenditure during walking (Carey & Crompton, 2005; Foster, Raichlen, & Pontzer, 2013). Our finding that individuals used more flexed lower limb joints at midstance during forest walking agrees with ethnographic observations of forest-dwelling people (Evans, 1937; Garvan, 1964; Schebesta, 1928; Turnbull, 1986), but is inconsistent with some laboratory-based studies that found no major difference in hip and knee joint posture at midstance between walking on even and uneven surfaces (Gates et al., 2012; Voloshina et al., 2013). Studies of walking on compliant and slippery surfaces have found that people tend to use more crouched postures to lower the body's centre of mass, potentially to help avoid falls by reducing the moment arm between the centre of mass and ground reaction forces (MacLellan & Patla, 2006; Marigold & Patla, 2002). A similar strategy may have been adopted by the participants in this study as means to avoid falls with obstacles on the ground of the forest understory that could cause tripping, but further research is necessary to test this possibility. Additionally, the use of more crouched legs could be another strategy alongside trunk inclination for avoiding contact with low branches in dense forest. Notably, we found that taller participants tended to use more flexed hip and knee joints, as well as more inclined trunks, during forest walking. This result suggests that shorter individuals may need to make fewer adjustments to their standard walking kinematics in forests, which could provide an additional selective force for reduced stature in forest-dwelling populations alongside reducing constraints on step length (Venkataraman et al., 2018).

The use of greater maximum foot heights during the swing phase in the forest condition is almost certainly a strategy to avoid tripping on obstacles. This finding concords with longstanding ethnographic observations of forest-living human populations (Evans, 1937; Garvan, 1964; Schebesta, 1928; Turnbull, 1986), as well as several laboratory-based studies that have reported a higher minimum toe clearance during walking on uneven surfaces (Gates et al., 2012; Schulz, 2011). Walking under these conditions therefore elicits a gait in which the foot is raised higher and thus the toe is probably further from the ground surface, which decreases the likelihood of it colliding with an obstacle as the leg is swinging forward. Increasing foot height involves greater lower limb joint flexion (Schulz, 2011), which probably requires more muscle activation and therefore higher energy expenditure (Kowalski et al., 2021). A goal of future research should be to determine whether maximum foot height is modulated dynamically while walking through complex terrain, as opposed to employing a conservative ‘high-stepping’ strategy at all times to avoid potentially severe injury costs.

Altogether, the kinematic adjustments made during forest walking that we observed suggest major consequences for walking economy on natural terrains. Without measurements of metabolic energy consumption we cannot determine how much forest walking would increase energy costs compared with walking in the open field. However, the difference would probably be greater than the 24% increase in cost of transport measured by Kowalsky et al. (2021) for participants walking on woodchips vs. a dirt path, because avoiding natural obstacles and maintaining balance in the forest understory would probably require more substantial adjustments to walking kinematics than does walking on woodchips. Cost differences could be closer to the range of the 50–115% increases previously measured for heavy brush and rocky terrains vs. even, hard surfaces (Soule & Goldman, 1972; Gast et al., 2019). Even differences at the low end of this range would have major energetic consequences for humans who must travel through challenging natural terrains during foraging. Given that both adult Tsimane men and women travel substantial distances during the day (averages: men, 9.9 km; women, 7.6 km; Davis, Gurven, Cashdan, in press), adjustments to walking kinematics in the forest probably result in consequential increases in active energy expenditure relative to what would be predicted from walking costs measured on even surfaces following standard procedures. Individuals from other subsistence-level societies who must forage over long distances on various challenging surfaces (e.g. brushland, rocky terrain, snow/ice) probably face similar energetic penalties owing to adjusted kinematics, and such costs should be considered in estimates of daily energy expenditure related to walking.

This study suggests that one important means by which humans can mitigate the potentially costly kinematic adjustments necessary when walking on challenging natural terrains is through the creation of trails. Trail walking was kinematically intermediate between open field and unbroken forest walking in some respects, including maximum foot height and midstance thigh angle, but was similar to open walking in terms of foot strike angle, trunk inclination and midstance knee angle. These results show that, by creating and utilising trails, humans may be able to walk in ways that are kinematically similar to walking on flat, even surfaces, and thus may achieve greater walking economy. The creation and maintenance of trails thus represents a good example of human niche construction. The Tsimane treat trails like a public good, with older individuals and village work parties actively maintaining the condition of trails for the good of the community. Tsimane individuals will sometimes make use of animal-made trails when they are foraging and no human-made trail is available. However, individuals will often use machetes to clear vegetation on animal trails for easier walking, and our personal experience is that walking on animal trails is almost as challenging as walking through unbroken forest, although more research is needed. Indeed, despite its potential significance in human foraging, trail creation and use have received almost no study beyond the dissertation research of Laden (1992), who worked with Efe hunter–gatherers and Lese farmers in the Ituri forest in the Democratic Republic of the Congo. Laden observed that travelling a given distance off trail takes twice as long as when on human-made trails, and suggested three important functions of the trail–territory system: connection between important places; placement in important habitat to allow access to hunting areas; and minimising the difficulty of travel and navigation. Similar observations have been made for the Mbendjele hunter–gatherers, also of the Congo Rainforest (Jang et al., 2019). Further research should explore how trails are created and maintained in the broader ‘energetic landscape’ of subsistence societies, how they contribute to cultural practices and how walking on human-made trails contrasts with walking on animal-made trails.

While the results of this study provide an interesting, preliminary picture of the kinematic adjustments that are necessary when walking in unbroken forest, we were limited in the range of surfaces we could investigate and did not quantify mechanical properties or other terrain characteristics such as obstacle height in the forest. Additionally, this study was limited in at least three major ways by our reliance on video cameras to obtain kinematic data: First, we were restricted to capturing motion from a single, short (~2 metres) segment of the forest transect, and therefore could only assess kinematic adjustment in response to the particular obstacles/impediments in this segment. While we tried to select a segment that was representative of the transect overall and did not include any large obstacles or footing hazards that would have required substantial deviations from steady gait dynamics, we recognise that no short segment could truly represent an ‘average’ stretch of forest. This limitation is highlighted by our finding of greater stride lengths in the forest relative to the open field, which stands in contrast to the shorter *average* step lengths over the full transect that we found in our previous study of forest walking (Venkataraman et al., 2018). The likely explanation for this discrepancy is that participants took longer steps to avoid small impediments in the particular segment of the transect that we recorded for this study. This scenario would be consistent with the findings of Matthis et al. (2018), who observed shorter steps on average but greater step length variability in people walking on rocky terrain compared with even terrain. Indeed, in any ‘natural’ terrain no single short segment that can be captured on video will provide a representation of the full diversity of surface characteristics that could require alterations in gait kinematics. Step-to-step variability is known to increase walking costs (O'Connor, Xu, & Kuo, 2012), and thus is another factor that could make walking on natural terrains such as those in forests more energetically expensive.

The second major limitation of our video-based motion capture was that we were forced to remove many recorded steps in the forest from our analysis owing to foliage obstructing markers. One consequence of this limitation was that we could not quantify kinematic variability between steps for a given terrain type, and thus could not compare the relative amounts of variability caused by walking in terrains like unbroken forest, which we would expect to be high based on previous studies of natural irregular terrains (e.g. Matthis et al., 2018). Marker obstruction also prevents the measurement of continuous kinematics across a stride, and so within-stride variation also cannot be assessed. While the problem of marker obstruction was particularly significant in the forest, even more open natural terrains often include obstacles like rocks and vegetation that can impede camera views of foot markers, preventing the measurement of many kinematic variables.

Finally, and relatedly, we were limited to a single camera view, and therefore could not capture three-dimensional kinematics. Thus, we were restricted to measuring sagittal plane motion in this study. The use of multiple cameras to capture 3D kinematics is certainly possible in natural settings, but it is logistically much more challenging than obtaining a single camera view, especially owing to the necessity of finding multiple camera positions that allow unobstructed views of all markers. A related problem that we encountered was difficulty in establishing a real-world coordinate system using a single camera. We set our camera views parallel to the walking route marked by flags to define an *x*-axis that was parallel to the ground, but it is possible that slight offsets in the angle of the camera view relative to the ground caused errors in some of our measurements. These errors should not have been systematic across conditions, and they are likely to have been small, since offsets between camera view and walking route were on average 0.9° in a subset of trials (*N* = 18) where we could calculate the offset angle. Nevertheless, given these different limitations, we emphasise that our findings are preliminary, and that further research using more sophisticated equipment is needed to study walking over longer distances and to quantify more kinematic variables to further characterise gait adjustments on different terrains.

One particularly promising technique for future investigations of gait in natural environments is the use of inertial measurement unit (IMU) technology. IMUs are small, light, wireless sensors containing a combination of motion-detecting features including an accelerometer, a magnetometer and a gyroscope. The data from these features provide information about sensor position, orientation and acceleration. Data from multiple IMUs placed at specific locations on the body can be used to quantify 3D limb segment and joint motion when following standardised data collection protocols and data processing routines. Validation studies have indicated that IMU systems produce reasonably accurate measurements of 3D kinematics during walking when compared with camera-based optical motion capture systems (Al-Amri et al., 2018; Cho et al., 2018). IMU systems can therefore be used to collect continuous 3D kinematic data under circumstances where camera-based motion capture is not feasible, and have already been applied to measure motion outside of the laboratory during activities such as marathon running (Reenalda, Maartens, Homan, & Buurke, 2016). To our knowledge, only Matthis et al. (2018) and Kowalsky et al. (2021) have used IMUs to record walking on complex natural terrains outdoors, and neither report joint kinematics in their study. Our future research with Tsimane and other subsistence societies will therefore employ technology like IMUs to better understand kinematic adjustments to walking on complex natural terrains, particularly in people who are accustomed to walking on these surfaces as part of daily activities such as foraging, and who do so barefoot or in minimal footwear. 3D kinematic data will allow researchers to explore other aspects of walking kinematics that could further affect economy and balance, such as step width and centre of mass motion (Donelan, Kram, & Kuo, 2001; Marigold & Patla, 2008; Thompson et al., 2018). Pairing such data with the use of electromyography to measure muscle activation and respirometry to measure energy use will help us understand how our species is capable of travelling on a wide diversity of challenging terrains, including the associated tradeoffs between walking economy and stability.

## Conclusions

Upright bipedal walking has long been argued to represent an adaptation for greater walking economy (Rodman & McHenry, 1980), as human bipedal walking is considerably less expensive than quadrupedal or bipedal walking in other primates (Nakatsukasa et al., 2004; Sockol et al., 2007). These findings have led some to argue that the evolution of economical bipedalism could have helped facilitate the initial adoption of hunting and gathering foraging strategies (Leonard & Robertson, 1997b; Kraft et al., 2021). However, the data presented here and in previous studies suggest that some of the kinematic features that are believed to give humans their edge in walking economy, such as use of extended legs and heel strikes (Pontzer & Kamilar, 2009; Webber & Raichlen, 2016), are compromised on irregular natural terrains. While other primates probably also need to adjust walking kinematics owing to surface irregularity, bipedal hominins could face especially high energetic penalties on irregular natural terrains if they cannot use the unique kinematic strategies that are critical to the energetic advantages of human bipedalism. If this hypothesis is true, reconstructions of hominin locomotor energetics that use standard laboratory-based measures of human walking costs may be underestimating the actual energy expenditure necessary for foraging and overestimating the energetic advantages of human bipedalism. This scenario has important implications for theories that relate hominin ecology to the evolution of economical bipedalism, as well as for the energy requirements of foraging in modern humans who rely on long daily travel distances for subsistence, such as hunter–gatherers. Further research is necessary to understand both the energetics of walking on natural terrains and the kinematic strategies humans use to maintain balance on such surfaces. Humans and other animals develop a repertoire of flexible motor-skills based on the challenges they encounter in their environments (Adolph & Young, 2021), and thus developmental experience travelling over difficult terrains may be critical to fostering kinematic strategies that optimise multiple aspects of locomotor performance, including safety, speed and economy. Thus, by studying locomotion in a diversity of human populations, especially those who regularly travel through complex natural environments, we may better understand the evolution of bipedalism.

## Data sharing statement

All data collected for this study and software used in analysis are available upon reasonable request.
